# Complications Rate and Related Factors After Laparoscopic Sphincter-Preserving Total Mesorectal Excision for Low Rectal Cancer: A Single-Center Study in Vietnam

**DOI:** 10.7759/cureus.60734

**Published:** 2024-05-21

**Authors:** Ly Huu Phu, Ho Tat Bang, Ung Van Viet, Hoang Danh Tan, Nguyen Trung Tin

**Affiliations:** 1 Gastro-Intestinal Surgery Department, University Medical Center Ho Chi Minh City, University of Medicine and Pharmacy at Ho Chi Minh City, Ho Chi Minh City, VNM; 2 Thoracic and Vascular Department, University Medical Center Ho Chi Minh City, University of Medicine and Pharmacy at Ho Chi Minh City, Ho Chi Minh City, VNM; 3 Department of Health Organization and Management, Faculty of Public Health, University of Medicine and Pharmacy at Ho Chi Minh City, Ho Chi Minh City, VNM; 4 Department of General Surgery, Faculty of Medicine, University of Medicine and Pharmacy at Ho Chi Minh City, Ho Chi Minh City, VNM; 5 Proctology Department, University Medical Center Ho Chi Minh City, University of Medicine and Pharmacy at Ho Chi Minh City, Ho Chi Minh City, VNM

**Keywords:** vietnam, related factors, complications, rectal cancer, sphincter-preserving surgery

## Abstract

Background: Colorectal cancer is a significant health concern. Surgery remains a critical component of the multimodal treatment strategy. The laparoscopic sphincter-preserving total mesorectal excision (TME) is increasingly utilized and effective, offering enhanced quality of life for patients compared to previous traditional methods.

Objectives: This study aims to determine the rate of complications and the related factors associated with complications following laparoscopic sphincter-preserving total mesorectal excision for low rectal cancer.

Methods: This retrospective study was conducted at the University Medical Center of Ho Chi Minh City from March 2022 to March 2023. It included patients aged 18 years and older diagnosed with low rectal cancer who underwent laparoscopic sphincter-preserving total mesorectal excision. Data on patient demographics, surgical details, and postoperative complications were retrospectively collected and analyzed. Follow-ups were conducted up to six months after surgery.

Results: Of the 83 patients included, the postoperative complications rate was 14.5%. The complications observed included surgical wound infections (five cases), anastomotic leaks (five cases, including three recto-vaginal fistulas and two pelvic abscesses), urinary retention (one case), and pneumonia (one case). A significant finding was the higher rate of distant metastases in patients with complications compared to those without (p=0.033).

Conclusion: Laparoscopic sphincter-preserving total mesorectal excision for low rectal cancer is safe and effective, with a high success rate and low complication rate during or after surgery. Anastomotic leakage remains the most significant complication. Despite advancements in surgery, modern suturing tools, and preoperative patient optimization, complications are avoidable. Therefore, understanding the related factors and implementing preventive interventions is crucial.

## Introduction

Colorectal cancer ranks as the fourth most prevalent cancer in Vietnam, following breast, liver, and lung cancers. The total number of new cases in 2022 was 16,835 [1]. Various treatment methods have been developed and implemented; among these, rectal surgery involving the removal of the anal sphincter and the creation of a permanent colostomy, known as Miles surgery, is the conventional surgical technique for the treatment of low rectal cancer [2]. However, treatment outcomes can detrimentally impact patient quality of life due to the necessity for colostomies, episiotomies, and associated complications [3].

Laparoscopic sphincter-preserving total mesorectal excision (TME) has emerged as a technique for treating low rectal cancer, aiming to maintain sphincter integrity and thus preserve the patient's bowel function. This method notably improves sphincter preservation, with up to 90% of patients retaining sphincter function, thereby maintaining defecation functionality while ensuring oncological efficacy [4]. Despite its benefits, the procedure has challenges and potential complications, which can significantly affect patient outcomes and quality of life post-surgery. The complications included bleeding, rectal anastomotic leaks, and residual abscesses. These complications not only detrimentally impact the patient's quality of life but also diminish the effectiveness of the treatment and may lead to an early recurrence of the cancer [5-8]. Besides, the cost of treatment for complications is a huge burden for the patient's family [9].

Despite advancements in surgical techniques, loop suture technology, and preoperative patient optimization, complication rates remain high, ranging from 16.3% to 41%, with intestinal anastomotic leakage occurring at rates of 3% to 20% [10-14]. Consequently, identifying factors related to these complications is crucial for the success of sphincter-preserving surgeries. This study aims to determine the complication rate and the related factors associated with complications after laparoscopic sphincter-preserving resection for rectal cancer treatment.

## Materials and methods

Study settings and participants

This retrospective study was conducted at the Department of Gastrointestinal Surgery, University Medical Center, Ho Chi Minh City, Vietnam. Since 2010, our center has implemented a standardized treatment protocol for rectal cancer, continuously updated in line with guidelines from the National Comprehensive Cancer Network (NCCN), the European Society for Medical Oncology (ESMO), and the Japanese Society for Cancer of the Colon and Rectum (JSCCR). All rectal cancer cases are evaluated and treated based on decisions made by the Multimodal Diagnostic Council.

Participants

Patients aged 18 years or older diagnosed with low rectal cancer were included in this study. Specific inclusion criteria based on tumor staging included: (1) low rectal tumors classified as T1 to T3 with a negative mesorectal fascia (MRF) and no evidence of extra mesorectal lymph node metastasis were considered for direct sphincter-preserving surgery. (2) Tumors staged greater than T3, MRF positive, or those with lateral pelvic lymph node involvement or unresectable features underwent preoperative chemoradiotherapy. These patients were re-staged using imaging studies, including MRI and post-therapy, to assess the response and plan subsequent surgical interventions. If post-therapy imaging showed no residual positive lateral lymph nodes, patients proceeded with sphincter-preserving surgery. In cases where lateral lymph node disease persisted, a low anterior resection combined with lateral lymph node dissection was performed to manage the disease comprehensively. Patients indicated for abdominoperineal resection (Miles surgery) were excluded from this study to focus on sphincter-preserving approaches. The other exclusion criteria were patients who did not adhere to the prescribed postoperative follow-up schedule and those who received postoperative care at another healthcare facility.

Data Collection and Tools

Demographic and clinical characteristics: Age, gender, BMI, and comorbidities, The American Society of Anesthesiologists (ASA) physical status classification system is used to assess the physical status of patients before surgery.

Lifestyle factors: Data on smoking and alcohol consumption histories were collected.

Tumor characteristics: Tumor size and location are assessed via endoscopy and imaging (MRI, CT), TNM staging, and regional or distant metastases.

Biochemical markers:Levels of carcinoembryonic antigen (CEA), hemoglobin (HGB), albumin, and creatinine were studied.

Surgical details: The surgery types done are low anterior resection, ultralow anterior resection, and intersphincteric resection, depending on whether laparoscopic procedures were employed and the extent of lymphadenectomy. Anastomotic height from the anus, and the number of stapler cartridges utilized. The total duration of the surgery; the amount of blood lost during surgery.

Postoperative outcomes: Patients were monitored for immediate complications such as surgical wound infections, anastomotic leaks, general complications, and how these complications were managed.

Long-term monitoring: The patient was followed-up for six months post-surgery to assess recovery and manage any late postoperative complications. Complications were classified according to the Clavien-Dindo Classification [6].

Statistical analysis

The collected data were entered by EpiData Entry software (version 3.1, EpiData Association) and analyzed with Stata software (version 16.0, Stata Statistical Software: Release 16, StataCorp LLC, College Station, TX, USA). We used the chi-square test to analyze the relationship between complications and qualitative variables (replaced with Fisher's exact test in case there was at least a cell with a value of 0 or an expected value <5). Besides, we used the Wilcoxon rank sum test to explore the associations for quantitative variables. The difference was statistically significant when the p-value was <0.05.

Ethics in research

This study received ethical approval from the Ethics Council in Biomedical Research of the University of Medicine and Pharmacy of Ho Chi Minh City on March 10, 2022 (Decision No. 295/HĐĐĐ-ĐHYD).

## Results

Demographic and clinical characteristics are presented in Table 1. The study included 83 patients diagnosed with low rectal cancer undergoing laparoscopic sphincter-preserving total mesorectal excision. The mean age of the participants was 61.5 ± 11.2 years, with a majority (53 patients, 63.9%) aged 60 years or older. The study involved 45 males (54.2%) and 38 females (45.8%). Body mass index (BMI) assessments showed that most patients (73.5%) were within the normal range (18.5-24.9 kg/m^2^). All patients maintained adequate albumin (≥3.5 g/dL) and hemoglobin (≥9 g/dL) levels. Regarding risk factors, 22.9% (19 patients) of the patients were smokers, and 19.3% (16 patients) reported alcohol use. The majority of the patients (58 patients, 69.8%) were classified as ASA Grades I and II, and most had stage 3 and stage 4 (69 patients, 83.1%).

**Table 1:** Characteristics of patients (n=83) TNM: tumor-nodes-metastases; ASA: American Society of Anesthesiologists; BMI: body mass index; CEA: carcinoembryonic antigen

Characteristics	Frequency	Percentage (%)
Age(mean ± standard deviation)	61.5 ± 11.2	
Age group		
<60	30	36.1
≥60	53	63.9
Gender		
Male	45	54.2
Female	38	45.8
Comorbidities		
Yes	39	47.0
No	44	53.0
BMI		
<18.5	12	14.5
18.5–24.9	61	73.5
≥25	10	12.0
Albumin, g/dL		
<3.5	0	0
≥3.5	83	100.0
CEA, ng/mL		
<5	56	67.5
≥5	27	32.5
Hemoglobin, g/dL		
<9	0	0
≥9	83	100.0
Blood types		
A	16	19.3
B	23	27.7
AB	3	3.6
O	41	49.4
Cigarette smoking		
Yes	19	22.9
No	64	77.1
Alcohol use		
Yes	16	19.3
No	67	80.7
ASA grade		
Grade I	9	10.8
Grade II	49	59.0
Grade III	25	30.1
TNM stage		
1^st^ stage	6	7.2
2^nd^ stage	8	9.7
3^rd^ stage	67	80.7
4^th^ stage	2	2.4
Tumor height		
5 cm	31	37.3
6–10 cm	52	62.7
Neoadjuvant treatment		
Yes	20	24.1
No	63	75.9

Surgical characteristics are presented in Table 2. All patients underwent total mesorectal excision with the laparoscopic technique. Lymphadenectomy was performed in all cases, with 73.5% (61 patients) undergoing D3 and 26.5% (22 patients) undergoing D2 dissection. Splenic flexure mobilization was necessary in 32.5% (27 patients). The surgical procedure details revealed that the mean measurements for the lower and upper resection margins were 2.3 ± 0.7 cm and 12.9 ± 3.9 cm, respectively. Anastomotic sites were primarily positioned 4-5 cm from the anus in 56.6% of the cases (47 patients), and 42.2% had sites 2-3 cm from the anus (35 patients). Low anterior resection was the most common type of surgery (46 patients, 55.4%), followed by ultralow anterior resection (36 patients, 43.4%). A majority of patients required an ileostomy (70 patients, 84.3%). The average surgery duration was 186.9 ± 44.7 minutes, with a mean blood loss of 35.5 ± 26.8 ml.

**Table 2:** Surgical characteristics (n=83) ^##^Mean ± standard deviation (min-max)

Characteristics	Frequency	Percentage (%)
Lymphadenectomy		
D2	22	26.5
D3	61	73.5
Splenic flexure mobilization	27	32.5
Lower resection margins (cm)	2.3 ± 0.7 (1–5)^ ##^	
Upper resection margins (cm)	12.9 ± 3.9 (10–30)^ ##^	
Stapler cartridges		
1	15	18.1
2	63	75.9
≥3	5	6.0
Anastomotic height		
4–5 cm from the anus	47	56.6
2–3 cm from the anus	35	42.2
Inter-sphincter-anal canal resection	1	1.2
Type of operation		
Low anterior resection	46	55.4
Ultralow anterior resection	36	43.4
Intersphincteric resection	1	1.2
Ileostomy	70	84.3
Tumor size (cm) mean ± standard deviation	4.48 ±1.56	
Surgery time (minutes)	186.9 ± 44.7 (90–300)^##^	
Blood loss (ml)	35.5 ± 26.8 (10–100)^ ##^	

Postoperative complications were reported in 12 patients (14.5%), detailed in Table 3, with five cases of surgical wound infection, five cases of anastomotic leakage, one instance of urinary disorders, and one case of pneumonia. Surgical wound infections were managed successfully with wound care and antibiotics, leading to recovery in all cases. Anastomotic leakage scenarios included complex treatments involving additional surgical interventions and prolonged recovery periods. No intra-abdominal infections, cardiovascular complications, bleeding, or deaths were reported. All complications were managed effectively with appropriate treatments.

**Table 3:** Description of postoperative complications MRI: magnetic resonance imaging; TME: total mesorectal excision

Complications	Number of case	Clavien-Dindo classification	Detail of complication
Total	12 cases (14.5%)		
Surgical wound infection	5 cases	Grade I (3 cases), grade II (2 cases)	There were 5 cases of surgical wound infection detected during the 1-month follow-up period after surgery. All of these cases recovered with wound care and antibiotic treatment.
Anastomotic leakage	5 cases	In this study, five cases of anastomotic leakage complications following laparoscopic sphincter-preserving total mesorectal excision for low rectal cancer were documented. Three female patients experienced rectovaginal fistulas, while two male patients suffered pelvic abscesses due to leaks at the anastomotic site.	
		Grade II	1. Male patient, aged 76: 21 days post-surgery, an MRI revealed a 7 cm × 6 cm × 5 cm fluid collection near the anastomotic site with a leakage at the 7 o'clock position. The patient was treated conservatively with antibiotics, leading to spontaneous resolution and ileostomy closure after three months.
		Grade IIIb	2. Male patient, aged 58: 14 days post-operation, this patient presented with a leak at the anastomotic site, leading to a localized pelvic abscess. He underwent drainage and suturing of the leak along with antibiotic therapy. Follow-up clinical examination and MRI after one month showed no recurrence of the leak. The ileostomy was closed five months later.
		Grade IIIb	3. Female patient, aged 69: this patient did not receive preoperative radiation therapy. Fourteen days following a low anterior resection, a rectovaginal fistula was detected. The leak was surgically repaired, and the patient was treated with antibiotics followed by adjuvant chemotherapy. The ileostomy was closed after six months.
		Grade IIIb	4. Female patient, aged 68: after receiving neoadjuvant chemoradiotherapy and undergoing TME with ileostomy, she developed a rectovaginal fistula approximately eight weeks post-ileostomy closure, during her second cycle of adjuvant chemotherapy. Surgical repair of the fistula was undertaken along with the creation of a transverse colostomy. Despite ongoing chemotherapy and follow-up, the fistula persisted three months later, but the patient declined further surgical intervention.
		Grade IIIb	5. Female patient, aged 49: following neoadjuvant chemoradiotherapy and TME with ileostomy, she experienced a vaginal perforation intraoperatively, which led to a rectovaginal fistula and pelvic abscess five days post-surgery. Initial conservative treatment with antibiotic irrigation was followed by surgical repair of the fistula one month later. Despite another surgical repair two months later, the fistula recurred ten days after ileostomy closure, necessitating the creation of a transverse colostomy and continued adjuvant chemotherapy. Six months later, the patient's condition worsened due to recurrent local cancer, leading to discontinuation of treatment cooperation.
Urinary disorders	1 case	Grade I	A case of bladder dysfunction after surgery. This case recovers through rehabilitation.
Pneumonia	1 case	Grade II	Pneumonia was discovered after surgery, treatment was stabilized with antibiotics, the patient recovered and was discharged without sequelae.

A statistical analysis was conducted to explore associations between various factors and postoperative complications (Table 4). No statistically significant differences were found between the groups with and without complications regarding age, gender, body mass index, chronic diseases, American Society of Anesthesiologists classification, adjuvant treatment before surgery, and other clinical parameters. The analysis indicated that distant metastases detected via computed tomography scanners were more prevalent in patients with complications (p=0.033).

**Table 4:** Factors related to postoperative complications (n=83) ASA: American Society of Anesthesiologists; CEA: carcinoembryonic antigen; CT-scanner: computed tomography scanner; MRI: magnetic resonance imaging; cTNM: clinical tumour-node-metastasis *Fisher's exact test ^#^Wilcoxon rank sum test

Characteristics	Complications (N=12)	No complications (N=71)	p-value
Age (years)	65.1 ± 8.3	61.8 ± 11.6	0.492^#^
Gender			0.757
Male	7 (58.3)	38 (53.5)	
Female	5 (41.7)	33 (46.5)	
Body mass index (kg/m^2^)	20.3 ± 2.1	21.8 ± 2.7	0.058^#^
Chronic disease			0.821
Yes	6 (50.0)	33 (46.5)	
No	6 (50.0)	38 (53.5)	
ASA classification			0.379*
I	0 (0.0)	9 (12.7)	
II	7 (58.3)	42 (59.1)	
III	5 (41.7)	20 (28.2)	
Adjuvant treatment before surgery			>0.999*
Yes	3 (25.0)	17 (23.9)	
No	9 (75.0)	54 (76.1)	
Hemoglobin (g/L)	126.2 ± 13.7	130.1 ± 19.3	0.216^#^
Albumin (g/L)	39.2 ± 4.1	40.2 ± 3.6	0.488^#^
Creatinine (mg/dl)	0.88 ± 0.22	0.87 ± 0.22	0.777^#^
CEA (ng/mL) (median (interquartile range))	6.3 (2.8-17.8)	3.3 (1.7-6.7)	0.078^#^
Tumor location through endoscopy			>0.999*
Middle rectum (6–10 cm)	8 (66.7)	48 (67.6)	
Lower rectum (1–5 cm)	4 (33.3)	23 (32.4)	
Perimeter			0.914*
0–25%	1 (8.3)	7 (9.9)	
25–50%	3 (25.0)	24 (33.8)	
50–75%	4 (33.3)	23 (32.4)	
≥75%	4 (33.3)	17 (23.9)	
Rectal stenosis			0.763*
Semi-rectal stenosis	6 (50.0)	38 (53.5)	
Nearly complete rectal stenosis	3 (25.0)	22 (31.0)	
Complete stenosis	3 (25.0)	11 (15.5)	
Tumor position MRI (n=82)			0.524*
Middle rectum (6–10 cm)	6 (50.0)	44 (62.9)	
Lower rectum (1–5 cm)	6 (50.0)	26 (37.1)	
Tumor invasive MRI (n=82)			0.405*
T1	0 (0.0)	1 (1.4)	
T2	0 (0.0)	8 (11.4)	
T3	10 (83.3)	40 (57.2)	
T4	2 (16.7)	21 (30.0)	
Regional lymph nodes MRI (n=82)			0.218*
N0	0 (0.0)	15 (21.4)	
N1	3 (25.0)	14 (20.0)	
N2	9 (75.0)	41 (58.6)	
Pelvic lymph nodes	0 (0.0)	0 (0.0)	
Distant metastases MRI (n=82)			0.146*
Yes	1 (8.3)	0 (0.0)	
No	11 (91.7)	70 (100.0)	
Tumor position CT-scanner (n=82)			>0.999*
Middle rectum	10 (83.3)	54 (77.1)	
Lower rectum	2 (16.7)	16 (22.9)	
Tumor invasive CT-scanner (n=82)			0.798*
T1	0 (0.0)	2 (2.9)	
T2	0 (0.0)	7 (10.0)	
T3	6 (50.0)	33 (47.1)	
T4	6 (50.0)	28 (40.0)	
Regional lymph nodes CT-scanner (n=82)			0.715*
N0	3 (25.0)	27 (38.6)	
N1	2 (16.7)	13 (18.6)	
N2	7 (58.3)	30 (42.8)	
Distant metastases CT-scanner (n=82)			0.033*
Yes	1 (8.3)	3 (4.3)	
No	9 (75.0)	66 (94.3)	
Suspect	2 (16.7)	1 (1.4)	
cTNM before surgery			0.388*
I	0 (0.0)	6 (8.4)	
II	1 (8.3)	7 (9.9)	
III	10 (83.3)	57 (80.3)	
IV	1 (8.3)	1 (1.4)	
Lymph node dissection			0.724*
D2	4 (33.3)	18 (25.3)	
D3	8 (66.7)	53 (74.7)	
Splenic flexure mobilization			>0.999*
Yes	4 (33.3)	23 (32.4)	
No	8 (66.7)	48 (67.6)	
Stapler cartridges			0.428*
1	3 (25.0)	12 (16.9)	
2	8 (66.7)	55 (77.5)	
≥3	1 (8.3)	4 (5.6)	
Anastomotic height			0.083*
4–5 cm from the anus	4 (33.3)	43 (60.6)	
2–3 cm from the anus	7 (58.3)	27 (38.0)	
Inter-sphincter-anal canal resection	1 (8.3)	1 (1.4)	
Type of surgery			0.245*
Low anterior resection	4 (33.3)	42 (59.2)	
Ultra-low anterior resection	8 (66.7)	28 (39.4)	
Inter-sphincteric resection	0 (0.0)	1 (1.4)	
Ileostomy			>0.999*
Yes	10 (83.3)	60 (84.5)	
No	2 (16.7)	11 (15.5)	
Tumor size (cm)	4.8 ± 1.8	4.4 ± 1.5	0.433^#^
Surgery time (minutes)	193.8 ± 57.9	185.7 ± 42.5	0.799^#^
Blood loss (ml)	50 ± 33	33.1 ±25	0.051^#^

Further analysis focused on the specific factors associated with anastomotic leaks, as shown in Table 5. The study examined five cases of anastomotic leaks among the 83 patients. No statistically significant difference was found in age, gender, body mass index, presence of chronic diseases, American Society of Anesthesiologists classification, hemoglobin levels, albumin levels, creatinine levels, or carcinoembryonic antigen levels between patients who developed leaks and those who did not. Additionally, no significant associations were found with preoperative treatment modalities, tumor location, or other surgical details. This suggests that the anastomotic leaks were not strongly correlated with the demographic or clinical characteristics examined, indicating the complexity of the factors influencing this complication.

**Table 5:** Factors related to anastomotic leakage complications (n=83) ASA: American Society of Anesthesiologists; CEA: carcinoembryonic antigen; CT-scanner: computed tomography scanner; MRI: magnetic resonance imaging; cTNM: clinical tumor-node-metastasis *Fisher's exact test ^#^Wilcoxon rank sum test

Characteristics	Leak (N=5)	No leak (N=78)	p-value
Age (years)	64.4 ± 10.5	62.1 ± 11.3	0.639^#^
Gender			0.656*
Male	2 (40.0)	43 (55.1)	
Female	3 (60.0)	35 (44.9)	
BMI (kg/m^2^)	20.9 ± 2	21.6 ± 2.7	0.491^#^
Chronic disease			>0.999*
Yes	2 (40.0)	37 (47.4)	
No	3 (60.0)	41 (52.6)	
ASA classification			>0.999*
I	0 (0.0)	9 (11.5)	
II	3 (60.0)	46 (59.0)	
III	2 (40.0)	23 (29.5)	
Adjuvant treatment before surgery			0.590*
Yes	2 (40.0)	18 (23.1)	
No	3 (60.0)	60 (76.9)	
Hemoglobin (g/L)	124.8 ± 12.0	129.9 ± 19.0	0.301^#^
Albumin (g/L)	39.2 ± 1.7	40.1 ± 3.8	0.467^#^
Creatinine (mg/dl)	0.8 ± 0.16	0.87 ± 0.22	0.370^#^
CEA (ng/mL) (Median (interquartile range))	3.3 (2.3-7.9)	3.5 (1.7-7.6)	0.752^#^
Tumor location through endoscopy			>0.999*
Middle rectum (6-10 cm)	4 (80.0)	52 (66.7)	
Lower rectum (1-5 cm)	1 (20.0)	26 (33.3)	
Perimeter			0.911*
0–25%	0 (0.0)	8 (10.2)	
25–50%	1 (20.0)	26 (32.3)	
50–75%	2 (40.0)	25 (32.1)	
≥75%	2 (40.0)	19 (24.4)	
Rectal stenosis			0.840*
Semi-rectal stenosis	2 (40.0)	42 (53.8)	
Nearly complete rectal stenosis	2 (40.0)	23 (29.5)	
Complete stenosis	1 (20.0)	13 (16.7)	
Tumor position MRI (n=82)			0.374*
Middle rectum (6–10 cm)	2 (40.0)	48 (62.3)	
Lower rectum (1–5 cm)	3 (60.0)	29 (37.7)	
Tumor invasive MRI (n=82)			>0.999*
T1	0 (0.0)	1 (1.3)	
T2	0 (0.0)	8 (10.4)	
T3	4 (80.0)	46 (59.7)	
T4	1 (20.0)	22 (28.6)	
Regional lymph nodes MRI (n=82)			0.817*
N0	0 (0.0)	15 (19.5)	
N1	1 (20.0)	16 (20.8)	
N2	4 (80.0)	46 (59.7)	
Pelvic lymph nodes	0 (0.0)	0 (0.0)	
Distant metastases MRI (n=82)			>0.999*
Yes	0 (0.0)	1 (1.3)	
No	5 (100.0)	76 (98.7)	
Tumor position CT-scanner (n=82)			>0.999*
Middle rectum	4 (80.0)	60 (77.9)	
Lower rectum	1 (20.0)	17 (22.1)	
Tumor invasive CT-scanner (n=82)			0.812*
T1	0 (0.0)	2 (2.6)	
T2	0 (0.0)	7 (9.1)	
T3	2 (40.0)	37 (48.1)	
T4	3 (60.0)	31 (40.2)	
Regional lymph nodes CT-scanner (n=82)			0.841*
N0	1 (20.0)	29 (37.7)	
N1	1 (20.0)	14 (18.2)	
N2	3 (60.0)	34 (44.1)	
Distant metastases CT-scanner (n=82)			0.189*
Yes	0 (0.0)	4 (5.2)	
No	4 (80.0)	71 (92.2)	
Suspect	1 (20.0)	2 (2.6)	
cTNM before surgery			0.667*
I	0 (0.0)	6 (7.7)	
II	1 (20.0)	7 (9.0)	
III	4 (80.0)	63 (80.8)	
IV	0 (0.0)	2 (2.5)	
Lymph node dissection			0.605*
D2	2 (40.0)	20 (25.6)	
D3	3 (60.0)	58 (74.4)	
Splenic flexure mobilization			0.658*
Yes	2 (40.0)	25 (32.0)	
No	3 (60.0)	53 (68.0)	
Stapler cartridges			0.692*
1	0 (0.0)	15 (19.2)	
2	5 (100.0)	58 (74.4)	
≥3	0 (0)	5 (6.4)	
Anastomotic height			0.687*
4–5 cm from the anus	1 (33.3)	46 (57.5)	
2–3 cm from the anus	2 (66.7)	32 (40.0)	
Inter-sphincter-anal canal resection	0 (0.0)	2 (2.5)	
Type of surgery			0.671*
Low anterior resection	2 (40.0)	44 (56.4)	
Ultra-low anterior resection	3 (60.0)	33 (42.3)	
Intersphincteric resection	0 (0.0)	1 (1.3)	
Ileostomy			0.583*
Yes	4 (80.0)	66 (84.6)	
No	1 (20.0)	12 (15.4)	
Tumor size (cm)	4.5 ± 1.1	4.5 ± 1.6	0.886^#^
Surgery time (minutes)	155.4 ± 34.3	188.9 ± 44.7	0.078^#^
Blood loss (ml)	58 ± 40.2	34.1 ±25.4	0.136^#^

## Discussion

In sphincter-preserving surgery, complications are frequent and can have significant long-term effects [15]. In our study involving 83 patients undergoing rectal resection with TME, including those receiving neoadjuvant chemoradiotherapy, the postoperative complication rate was 14.5% (12 patients). This rate is comparatively lower than those reported in previous studies. A meta-analysis by Shirouzu et al. found complication rates ranging from 12.5% to 32.1% [10], while research by Watanabe et al. reported a general complication rate of 21.77% in Japan [3]. Research by Bediako-Bowan et al. [11] and Kanso et al. [16] noted even higher rates, at 41% and 47%, respectively. These earlier studies were conducted when surgical techniques were less refined than they are today. Furthermore, the improvement of perioperative care and the systematic implementation of enhanced recovery after surgery (ERAS) protocols played an important role. These protocols emphasize early mobilization, optimized pain management, and careful fluid management, which collectively reduce the likelihood of complications. Our study’s lower complication rate may also reflect a selective patient cohort with meticulous preoperative planning and postoperative management. These results emphasize the importance of continuous improvement in surgical practices and patient care protocols, which are crucial for minimizing the adverse outcomes associated with rectal cancer surgeries.

The mean age of our study participants was 61.5±11.2 years; 63.9% (53 patients) were aged 60 years or older, and 54.2% (45 patients) were male. Sixty-one patients (73.5%) had a body mass index within the normal range. Consistent with the literature, risk factors for complications included male gender, obesity, comorbid medical diseases, alcohol consumption, and albumin levels below 3.5 g/dL [3]. Other factors like family history of cancer, ASA classification, advanced age, nutritional status, and surgeon experience have also been implicated in higher complication risks [17]. While CEA is considered a marker for colorectal cancer, elevated CEA levels suggest the possibility of an invasion or spread of cancer. Our current data did not find a clear relationship between CEA levels and surgical complications [18].

Surgical area infection is the most common complication post-surgery, with occurrences ranging from 63% to 95% [10]. Of these, superficial surgical area infections (Clavien-Dindo levels I and II) are the most common, whereas deep surgical area infections or deep abscesses intra-abdominal (Clavien-Dindo grades III and IV) occur at a rate of about 0-15.3% [10,16,17]. In our study, five patients (6.03%) experienced superficial surgical wound infections. All these patients recovered with wound care and antibiotic therapy, and no deep surgical site infection cases were recorded.

Complications from colorectal anastomosis leaks are serious issues following sphincter preservation surgery. Previous reports indicate that the anastomotic leak rate ranges from 3% to 20%, significantly increasing the risk of postoperative death by up to 27%, as well as the risk of permanent colostomies and cancer recurrence [11,12,19,20]. Treatment of these complications has a high success rate but can still leave severe and lasting sequelae [6,7]. About 6% of our subjects who experienced leaks received individualized treatment. This highlights the importance of optimizing patient treatment before surgery and regularly implementing ERAS protocols from admission through post-operation. Furthermore, our practice of creating ileostomies in 84.3% of cases to protect the anastomosis significantly reduces the rate of anastomotic leakage and other serious complications [21-23]. The literature supports these results, with 100% of patients in certain studies receiving an ileostomy to protect the rectal anastomosis [16,17]. Shiomi et al. suggest that when the height of the rectal anastomosis is ≤5 cm from the anal verge, and particularly when ≤2 cm, opening a stoma to the skin is necessary [24].

The actual role of percutaneous ileostomy after rectal resection continues to be debated, presenting a challenge for surgeons due to complications related to the ileostomy status. The literature indicates that the rate of ileostomy after rectal resection ranges from 14% to 100% [10]. Furthermore, these patients must also undergo surgery to close the ileostomy, which carries many risks [22,25-27]. The research by Brisinda et al. showed that ileostomy did not necessarily help reduce the anastomotic leak rate [19].

Common serious medical complications, such as pneumonia, kidney failure, and cardiovascular complications, occur at a rate of about 1% [3,9,19]. Our study also recorded one patient with postoperative pneumonia who recovered with medical treatment and no cases of severe kidney or cardiovascular failure. In terms of ileostomy closure, 70 cases had an ileostomy opened to the skin, 69 cases were closed, and one case failed to close due to an early recurrence of cancer in the pelvis. There were 17 cases (24.6%) of late ileum closure, mostly due to patients receiving adjuvant chemotherapy post-surgery to avoid treatment interruption, followed by those experiencing complications from anastomotic leakage.

This study has several limitations. It was conducted at a single medical center, which may limit the generalizability of the results. Furthermore, the sample size of 83 patients, while sufficient for preliminary observations, is insufficient to apply the findings or establish definitive conclusions broadly. Additionally, the retrospective nature of data collection and the lack of comprehensive analysis of all potential risk factors could impact the accuracy and depth of the findings. Lastly, the relatively short follow-up period may not fully capture late postoperative complications or long-term outcomes.

## Conclusions

Laparoscopic sphincter-preserving total mesorectal excision for low rectal cancer is safe and effective, with a high success rate and low complication rate during or after surgery. Anastomotic leakage remains the most significant complication. Despite advancements in surgery, modern suturing tools, and preoperative patient optimization, complications are avoidable. Therefore, understanding the related factors and implementing preventive interventions is crucial.
